# Regenerative endodontic procedures for two traumatized mature anterior teeth with transverse root fractures

**DOI:** 10.1186/s12903-022-02152-y

**Published:** 2022-04-12

**Authors:** Jing Lu, Bill Kahler

**Affiliations:** 1 Fujian Key Laboratory of Oral Disease, School and Hospital of Stomatology, Fujian Medical University, Fuzhou, People’s Republic of China; 2 School of Dentistry, University of Queensland, 288 Herston Road, Herston, Brisbane, 4006 Australia

**Keywords:** Calcium hydroxide apexification, Bioceramic apical barrier techniques, Regenerative endodontic procedures, Transverse root fractures

## Abstract

**Background:**

Regenerative endodontic procedures (REPs) are an alternative treatment in immature traumatized teeth with necrotic pulp/apical periodontitis. However, this procedure has been infrequently reported in multiple transverse root fractures of mature teeth. This case report describes management of a patient with multiple horizontal root fractures in 2 maxillary central incisors that were successfully treated with REPs.

**Case presentation:**

A 17-year-old girl had a history of traumatic injury to mature teeth 11 and tooth 21 resulting in multiple transverse root fractures. Clinical examination showed that both teeth responded to electric and thermal pulp sensibility tests with prolonged severe pain and were tender to percussion and palpation. Periapical radiographic examination showed both teeth were fully developed and had multiple transverse fractures in the mid-root. The pulp diagnosis was consistent with symptomatic irreversible pulpitis. REPs were initiated with only the coronal fragments treated to preserve pulp vitality in the apical fragment for potential pulp tissue regeneration. After REPs, clinical signs/symptoms subsided, and the two teeth were followed for 48 months when cone beam computed tomography (CBCT) imaging was also undertaken. At the last review, the case demonstrated root fractures healing with calcified tissue and pulp calcification in the apical fragments. Both teeth were stable and in function.

**Conclusions:**

REPs have the potential to be used to treat traumatized and symptomatic mature permanent teeth that have sustained transverse root fractures.

## Background

Transverse root fractures are an uncommon injury. The incidence of pulp necrosis in a landmark study was 22% of 400 teeth that developed pulp necrosis [1]. In these instances, generally only the coronal fragment requires endodontic treatment [2–4]. A recent review advocated the use of several long-term calcium dressings to encourage the formation of a hard tissue barrier and a conventional root filling or placement of a bioceramic material as an apical barrier technique with the remainder of the canal filled with a conventional root filling [2]. Both approaches have shown good long-term outcomes [3, 4].

REPs have also been reported with successful outcomes for immature teeth with pulp necrosis following trauma [5]. The three primary goals of REPs are (1) resolution of signs and symptoms of infection, (2) further root maturation with increased root width, root length and apical closure, and a potential return of pulp sensibility responses suggesting a return of specialized tissue (AAE 2018) [6]. Regarding root fractured teeth, REPs could allow for restored homeostasis and provide a natural defense that may promote tooth survival not possible with conventional apexification approaches [7, 8]. There appear to be few reports where REPs have been used to successfully treat teeth with horizontal/transverse root fractures. Less frequently, REPs have been utilized for the treatment of mature teeth following trauma [9]. Arango-Gómez et al. reported on two immature teeth treated with REPs using platelet-rich plasma (PRP) which were reviewed for four years [10]. Intra-canal calcification was evident in both canals and healing was by interposition of bone and fibrous connective tissue which was confirmed by cone beam computed tomography (CBCT) imaging. Furthermore, CBCT imaging taken after treatment with REPs showed that there was separation of the fractured fragments which may have prejudiced the possibility of hard-tissue union [10]. Saoud et al. reported on a mature tooth with a transverse root fracture and healing appeared to be by interposition of connective tissue [11]. However, progressive healing by formation of hard tissue between the fractured fragments was noted at the 14- and 19-month review. Chaniotis treated a mature incisor that was root fractured using REPs utilizing a different protocol than that described by the American Association of Endodontists “Clinical considerations for a regenerative endodontic procedure” [6] by introducing a combination of blood and MTA to act as a scaffold to induce hard tissue repair of the root. The author suggested that it was hoped hard tissue healing could be achieved using REPs. However, in that case, healing was determined to be by interposition of connective tissue with no mineralized tissue detected [12].

The aim of this report is to describe the treatment of two mature teeth with transverse root fractures with REPs and their subsequent healing response.

## Case presentation

**Fig. 1:**
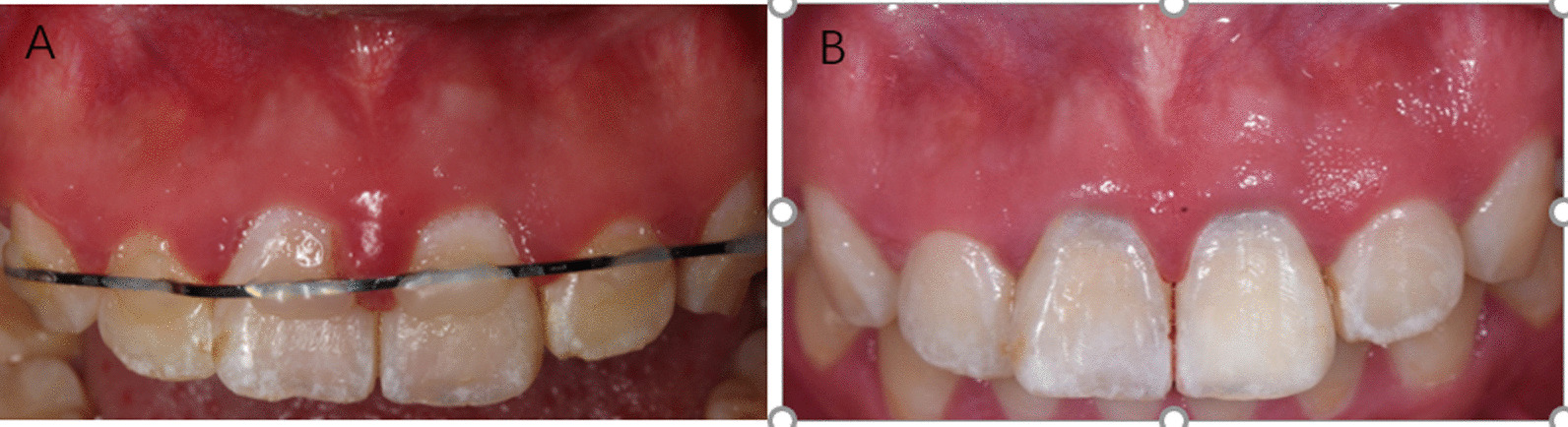
**A** Clinical photograph taken at the first visit after the teeth were splinted with a wire and composite resin splint. **B** Clinical photograph taken at the 48-month review showing minimal staining at the cervical margin of the crowns

**Fig. 2:**
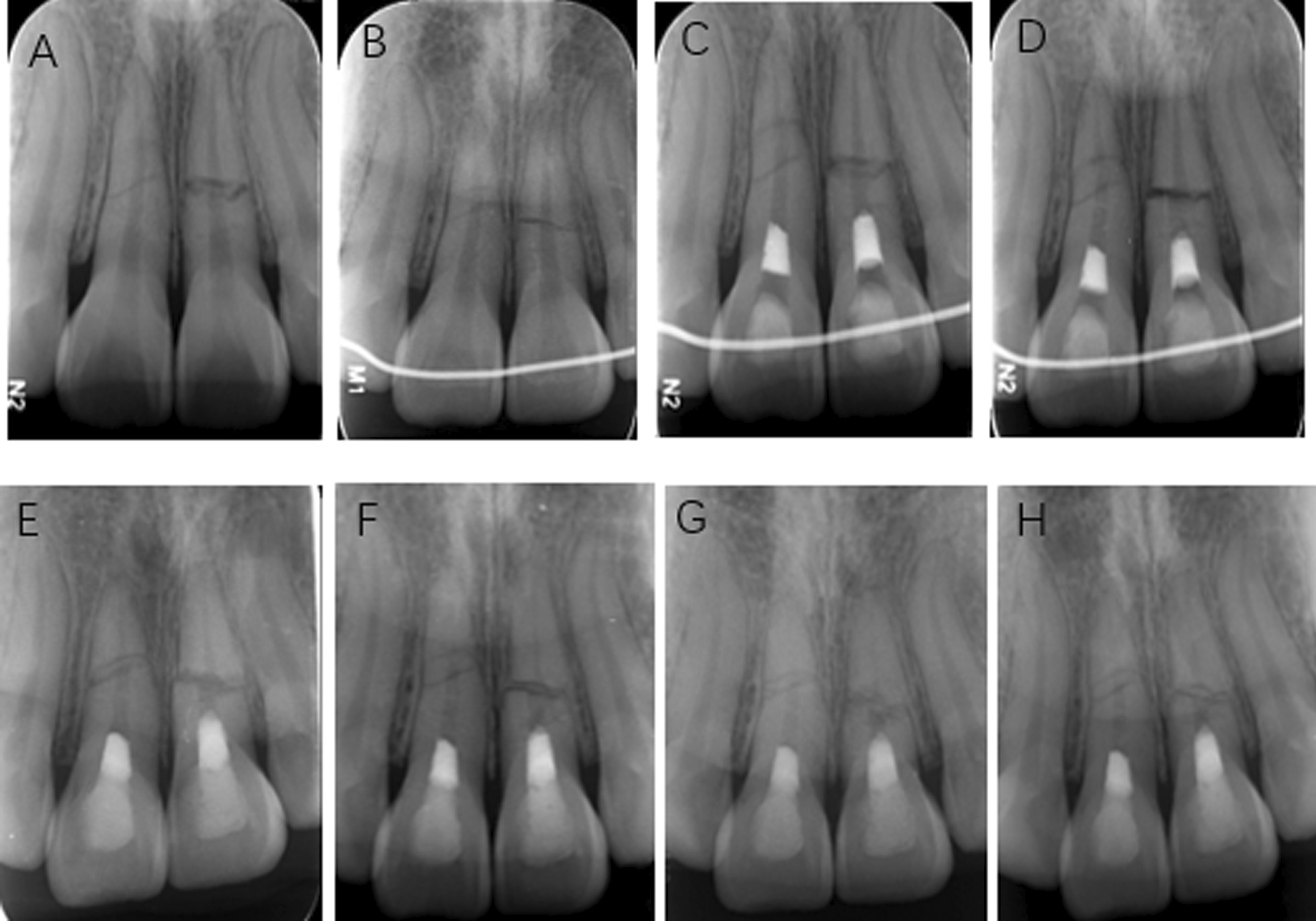
**A** Periapical radiograph of teeth #11 and #21 after trauma. The roots were completely formed. Multiple horizontal fractures in the middle third of the roots of teeth #8 and tooth #9 were evident. **B** Preoperative periapical radiograph before REPs. **C** Postoperative periapical radiograph after completion of REPs. **D** Postoperative periapical radiograph at the 1-month follow-up. No radiolucent lesion developed at the fracture line. **E** Postoperative periapical radiograph at the 6-month follow-up. Progressive healing of the horizontal root fracture by formation of hard tissue between fragments of tooth #21. **F** Postoperative periapical radiograph at the 14-month follow-up. More calcified in the fracture lines and root canal space of apical fragment of both teeth. **G** Postoperative periapical radiograph at the 25-month follow-up. Further healing of horizontal root fracture by formation of hard tissue, and calcification in the apical fragments of both teeth. **H** Postoperative periapical radiograph at the 36-month follow-up. The formation of calcified tissue continued between fragments of tooth #21 and coronal part of tooth #11. Partial canal obliteration is also evident in the coronal fragment. There is near complete calcification of the pulp space in the apical fragment. There was no evidence of a periapical lesion for either tooth

A 17-year-old girl suffered from a traumatic injury to her maxillary anterior teeth one month ago (Fig. 2A). The girl complained of severe pain to teeth 11 and 21 to touch and biting. Clinical examination showed that teeth 11 and 21 were slightly displaced labially (Fig. 1). The teeth responded to electric and cold pulp sensibility tests (tooth #11: 77/80, tooth #21: 56/80). The teeth were moderately tender to percussion and palpation. Both teeth showed grade II mobility. Periodontal probing was in the 2–3 mm range. Radiographic examination showed that teeth 11 and 21 had fully developed and demonstrated multiple transverse fractures in the middle third of the root of tooth 11 and tooth 21 (Fig. 2B). The diagnosis was multiple transverse root fractures with treatment was required due to persistent pain experienced by the patient despite splinting of the teeth for 1 month. Treatment options included root canal treatment of the coronal fragment of the root, REPs for the coronal fragment, and extraction. The patient decided to have REPs and informed consent was obtained.

At the first visit, teeth 13, 12, 11, 21, 22 and 23 were splinted for 4 weeks after the first visit. Local anesthesia with 2% lidocaine containing 1:100,000 epinephrine was administered. The teeth were isolated with a rubber dam. Under a surgical microscope (Zumax, Zuzhou, China), the canals were accessed, and a moderate amount of bleeding immediately drained through the access cavity. The canals were gently irrigated with 1.5% sodium hypochlorite solution to a level of a1mm coronal to the fracture. The working length of each tooth was extended up to the most coronal fracture line determined radiographically with a #25 hand K-file. The canals of the coronal fragments were carefully, sequentially debrided to #40 K-files with constant irrigation with sodium hypochlorite to the most coronal fracture line preserving vital pulp tissue and with only minimal to no filing of the canal walls. The canals of the coronal fragments were dried with paper points and dressed with calcium hydroxide (ApexCal; Ivoclar Vivadent AG, Schaan, Liechtenstein). The access cavity was temporized with a sterile cotton pellet and glass ionomer cement (Fuji IX, GC, Tokyo, Japan).

At the second treatment visit two weeks later, both teeth were asymptomatic. Local infiltration anesthesia with 3% Mepivacaine (Septodont, Taican, Jiangsu, China) without vasoconstrictor was administered. The teeth were isolated with a rubber dam and the access cavity reopened. Calcium hydroxide was removed with copious amounts of sodium hypochlorite irrigation followed by saline solution irrigation. The coronal canal was dried and rinsed with 17% EDTA solution (Langlishangwu, Wuhan, China) for 1 min in each tooth and dried again. A #40 hand K-file measuring 21 mm was used to gently penetrate the apical fragment of the canal containing vital pulp to induce bleeding into the coronal canal spaces up to the cemento-enamel junction. After a blood clot was formed, CollaCote (Integra Life Sciences, Shanghai, China) was placed over the blood clot in the canal of each tooth. A 3 mm thickness of iRoot BP (Innovative Bioceramix, Inc, Shanghai, China) paste was then placed against the CollaCote followed by a moist cotton pellet. The teeth were restored with glass ionomer cement and composite resin (Fuji IX, GC, Tokyo, Japan). A final radiograph was taken to verify the location of the coronal seal (Fig. 2C). The patient was reviewed after 1, 3, 6, 12, 24, 36, and 48 months, respectively.

## Follow-up examinations

At a 1-month follow-up (Fig. 2D), the patient was asymptomatic. Teeth 11 and 21 responded negatively to Endo-Ice, electric pulp tests (EPT), and no symptomatic response to percussion and palpation. Radiographic examination revealed was not remarkable. The splint was removed at this appointment.

At a 6-month follow-up (Fig. 2E), teeth 11 and 21 were asymptomatic. Radiographic examination revealed the transverse root fracture of tooth 21 was progressively healing by formation of hard tissue between fragments, while tooth 11 showed no change. The teeth did not respond to cold and EPT.

At a 14-month follow-up (Fig. 2F), the teeth were asymptomatic. Radiographic findings show that teeth 11 and 21 were more calcified in the fracture lines and the root canal space of apical fragment. Teeth had a delayed response to Endo-Ice, positive response to EPT in a comparable range and no symptomatic response to percussion and palpation.

At the 25-month follow-up (Fig. 2G), both teeth were asymptomatic. Radiographic examination demonstrated the process of further healing with calcified tissue in the fracture line of tooth 11. There was no presence of periapical pathology in either tooth. Teeth had positive response to Endo-Ice and EPT and no symptomatic response to percussion and palpation.

At the 36-month follow-up (Fig. 2H), both teeth were asymptomatic and functional. Radiographic findings showed that formation of calcified tissue continued between fragments of tooth #21. In both teeth, the apical fragments had been calcified, and the coronal fragment of tooth 11 had partially calcified. No displacement of the fractured fragments was confirmed. There was no evidence of periapical pathology. Teeth had positive response to Endo-Ice and EPT (and no symptomatic response to percussion and palpation. Tooth #21 exhibited grade I mobility, while tooth #11 had normal mobility.

**Fig. 3:**
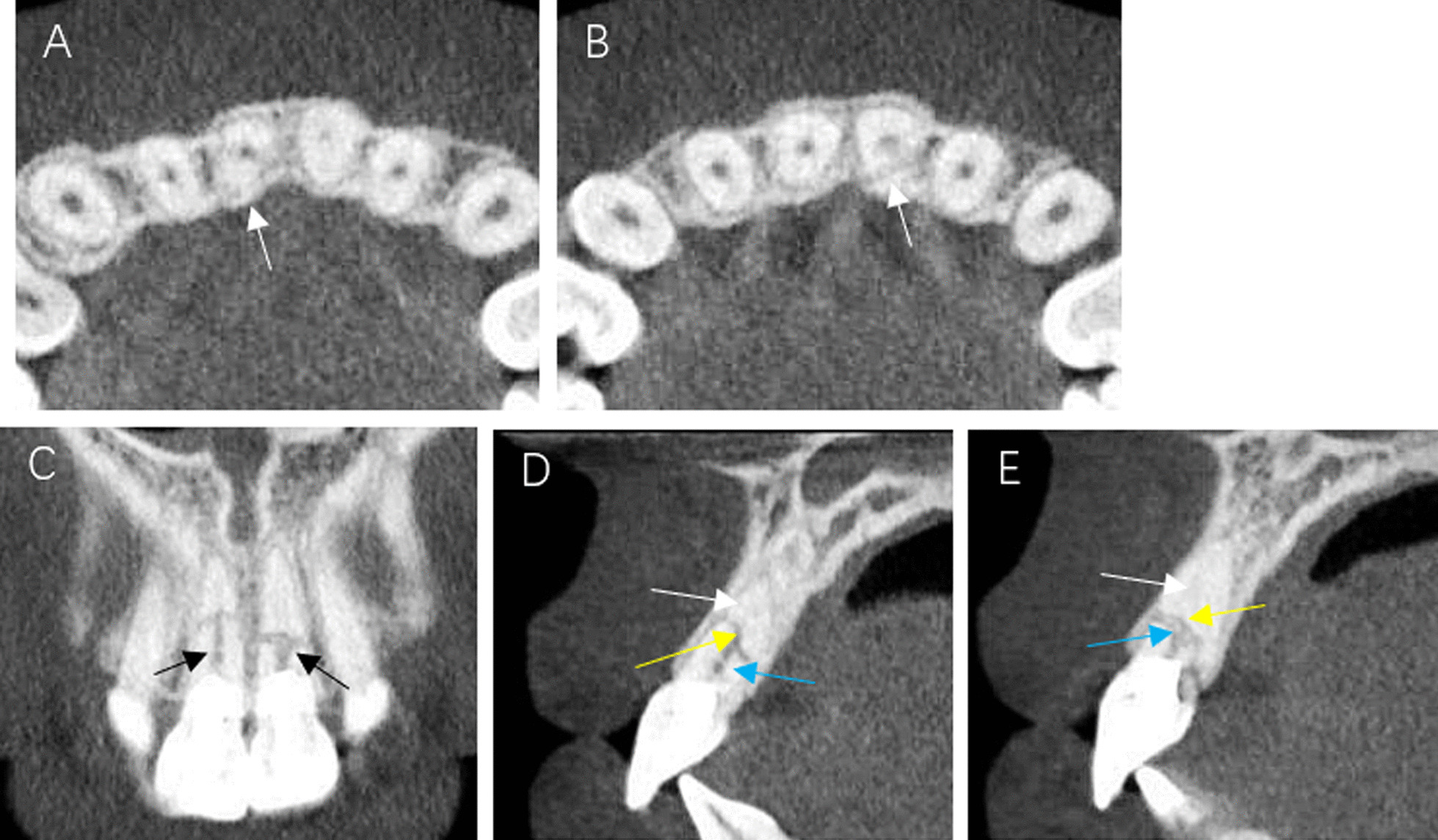
CBCT imaging of the root-fractured teeth taken at the 36-month review. **A** Axial review of tooth #11 **B** Axial review of tooth #21 **C** Coronal view Intracanal calcification in the coronal fragment is evident (black arrow) **D** Saggital view of tooth #11. Canal obliteration in the apical fragment is evident (white arrow) as is calcific tissue between the fractured fragments (yellow arrow). There is also mineralized tissue evident is the coronal fragment (blue arrow). **E** Saggital view of tooth #21 showing a similar calcific response to tooth #11

At the 48-month CBCT review, images appear to show healing by interposition of fibrous connective tissue with close apposition of the fragments (Fig. 3B). A clinical photograph reveals slight discolouration of the cervical margin for both treated teeth (Fig. 1B).

Chronological clinical and radiographic examinations were summarized in Table 1.

**Table 1:** Chronological clinical and radiographic examinations

Time	Tooth	Clinical examination				Radiographic examination	
		Cold testing	EPT	Percussion	Palpation	Size change of fracture lines	Periapical radiolucency
Preoperative	#11	–	77/80	+	+	N/A	–
	#21	–	56/80	+	+	N/A	–
1-month recall	#11	–	80/80	–	–	No change	–
	#21		80/80	–	–	No change	–
6-month recall	#11	–	80/80	–	–	Reduction	–
	#21	–	80/80	–	–	No change	–
14-month recall	#11	+	58/80	–	–	Reduction	–
	#21	+	69/80	–	–	Reduction	–
25-month recall	#11	+	31/80	–	–	Reduction	–
	#21	+	56/80	–	–	Reduction	–
36-month recall	#11	+	12/80	–	–	Reduction	–
	#21	+	23/80	–	–	Reduction	–
48-month recall	#11	+	20/80	–	–	Reduction	–
	#21	+	16/80	–	–	Reduction	–

## Discussion and conclusions

In a landmark study of 400 transverse root fractured teeth, healing was determined to be by hard tissue fusion of the fragments (30%), interposition of periodontal ligament (PDL) (43%), interposition of PDL and bone (5%) and non-healing with pulp necrosis and inflammatory changes between the fragments (22%) [1]. This study reported that a young age, an immature tooth, a positive response to pulp sensibility at the time of injury, mobility of the coronal fragment and close apposition of the fractured fragments were important factors for pulp healing and hard tissue repair of the fractured fragments. In the current study, a diagnosis of symptomatic irreversible pulpitis required endodontic treatment had to be undertaken. The differences in the current report, with the aforementioned landmark study, was that this was a mature tooth which at the time of the injury the coronal fragment had Grade II mobility and an acute response to pulp sensibility testing. However, as REPs were the chosen treatment option, healing by interposition of PDL was achieved (Figs. 2F and 3). Tooth 21 healed with a mix of calcific tissue and connective tissue with connective tissue predominating in tooth 11. Hard tissue healing of the fractured segments was not shown.

Both teeth were diagnosed with irreversible pulpitis and an attempt was made to preserve vital pulp tissue at the first appointment. This followed the approach for the treatment of a root fractured mature incisor tooth described by Saoud et al. [11]. Jung et al. also described remaining residual pulp tissue in five of nine treated premolar teeth [13]. However, in another report, two immature teeth treated with root fractures were diagnosed with pulp necrosis and a sinus tract [10].

Heithersay and Kahler in a histological report of two cases of transverse root fractures showed healing involved both pulp and periodontal tissues [14]. In their report, one case healed by hard tissue healing and the other by a fibrous union. In the current report, endodontics was initiated after the teeth were diagnosed with irreversible pulpitis. An advantage of treating the root-fractured teeth with REPs may be that introducing a blood clot into the coronal fragment may allow a further avenue of repair by angiogenesis as compared to the traditional approaches of apexification where the coronal fragment is completely debrided with no remaining vital tissue.

Current modalities for treatment of permanent teeth with transverse root fractures include calcium hydroxide dressings or apical barrier techniques with placement of a bioceramic. These approaches are generally successful. New treatment approaches should be equal or offer improved outcomes if the new approach is to be adopted [8]. In the current report, both teeth were responsive to cold and EPT evident at the 6-month through to a 48-month recall. Therefore, the use of REPs achieved an outcome potentially superior to current approaches for treatment of teeth with transverse root fractures. In a root fractured tooth, the apical fragment is vital and therefore has an adequate vasculature [15]. Therefore, invoking bleeding to promote healing under aseptic conditions is unlikely to be a concern to the ongoing vitality of the apical fragment, as evidenced by this report where successful healing occurred in two root-fractured mature teeth. However, the potential for damage to the vasculature of the apical fragment is acknowledged so further reports are required to validate this approach.

In the current report, pulp canal obliteration (PCO) could be observed in both the coronal and apical fragment. Andreasen et al. reported that PCO always followed revascularization of an uninjured pulp [16]. These authors also suggested that PCO could be a response to an additional trauma. Certainly, invoking bleeding must be considered an additional trauma. However, calcification of the apical fragment is a common finding in root-fractured teeth [15, 17]. However, the current report also showed calcification of the coronal fragment which demonstrates the presence of vital tissue that allowed deposition of mineralized tissue (Figs. 2F, 3C, D). The true nature of that tissue is unknown as this can only be determined by the gold standard of histological examination. It is known that REPs have been shown to be a reparative process involving stem cells of the apical papilla and mesenchymal cells from the periodontium and bone [18]. The origin and cellular lineage of the reparative processes in this case are unknown.

The risk of discolouration of teeth treated with REPs is associated with medicaments especially minocycline when used in a tri-antibiotic paste and MTA which is used as an intracanal barrier [19]. In the current report, calcium hydroxide was used to disinfect the canal rather than tri-antibiotic paste. Calcium hydroxide has been shown to result in significantly less discolouration of teeth when used as a medicament in REPs [20]. Furthermore, iRoot BP (Innovative Bioceramix, Inc, Shanghai, China) was used as an intracanal barrier over the induced blood clot instead of MTA. In the current report, there was minimal staining of the crown at the cervical aspect of the crown (Fig. 3B). The authors are unaware of any prior report on potential discolouration of teeth when iRoot is used but a favourable result is seen in the presented case.

A recent review advocated that long-term dressing with calcium hydroxide is the preferred treatment for transverse root fractures that require endodontic treatment [2]. It was suggested that calcium hydroxide apexification was a simpler technique than bioceramic apical barrier techniques and that the long-term use of calcium hydroxide obtains adequate antibacterial activity. It was further argued that bioceramic apical barrier techniques were technically more difficult with the risk of extrusion into the tissues between the two fractured fragments [2]. Both techniques have favorable outcomes in the treatment of root-fractured teeth when the coronal fragment is infected [2, 4]. However, both techniques also involve complete removal of tissue from the coronal fragment and therefore not allow a biological response of repair. In a time in our profession when regenerative endodontic procedures are becoming more widely accepted, it may be time to consider a third treatment option involving REPs in the treatment of root-fractured teeth.

This report demonstrates a novel approach in the treatment of root fractured teeth using REPS on two mature teeth where healing by interposition od PDL was shown. REPs are being increasingly used in the treatment of immature teeth that have sustained trauma as there is the potential for further root development and a return of neurogenesis. However, hard tissue union following REPs was not reported This report illustrates that REPs can also be used in the treatment of root-fractured mature teeth. Further clinical studies with larger sample sizes are required.

## Data Availability

Data was not made available due to patient confidentiality.

## Abbreviations

REPs: Regenerative endodontic protocols
CBCT: Cone beam computed tomography
EPT: Electric pulp testing
